# Bridging DSM/ICD and Developmental Neuroscience: A Neurocognitive Profile Specifier Framework for Neurodevelopmental Disorders

**DOI:** 10.1192/bjo.2026.11318

**Published:** 2026-06-30

**Authors:** Mariana Galvao de Oliveira

**Affiliations:** South London and Maudsley NHS Foundation Trust, London, United Kingdom. King’s College London, London, United Kingdom

## Abstract

**Aims:**

Diagnostic systems for neurodevelopmental disorders, including DSM–5–TR and ICD–11, provide essential categorical classifications for clinical communication, service eligibility, and care planning. However, these systems offer limited capacity to explain the marked heterogeneity observed within diagnostic groups or to link diagnosis meaningfully to underlying neurocognitive mechanisms. In parallel, dimensional frameworks grounded in developmental neuroscience have generated substantial mechanistic insight yet remain difficult to operationalise in routine clinical practice. This gap between descriptive diagnosis and mechanistic understanding limits formulation, training, and translational consistency across clinical settings.

**Aim**

To propose a neurocognitive profile specifier framework that integrates developmental neuroscience constructs into DSM/ICD-based diagnosis using a shared, clinically usable language applicable across education, clinical practice, and service contexts.

**Methods:**

This conceptual framework defines five neurocognitive profile domains consistently implicated in neurodevelopmental conditions: Social Cognition; Executive Function and Cognitive Flexibility; Reward and Motivation Sensitivity; Sensory Processing; and Emotional Regulation. Each domain is characterised using concise, developmentallyinformed specifiers (e.g. intact, variable, markedly impaired), derived from information routinely obtained during neurodevelopmental assessment, including clinical interview, collateral history, and standardised measures interpreted relative to developmental norms. The framework is designed to be applied following categorical diagnosis, preserving existing descriptive specifiers while adding a mechanistically coherent profile layer to clinical formulation.

**Results:**

The framework produces a concise neurocognitive profile embedded within the diagnostic formulation. Clinically, this supports clearer intra-diagnostic differentiation, improves multidisciplinary communication, and facilitates targeted intervention planning without reliance on biological markers not yet suitable for routine use. Across services and training settings, it provides a structured approach for teaching formulation and linking assessment findings to intervention strategies. In addition, the use of clinically familiar but mechanistically grounded domains facilitates alignment with neuroscience-informed research, supporting cumulative and translational understanding.

**Conclusion:**

Neurocognitive profile specifiers offer a pragmatic means of aligning diagnostic practice with developmental neuroscience while preserving categorical classification. By contextualising diagnosis rather than replacing it, this framework supports more precise formulation, enhances educational clarity, and provides a scalable approach to personalised care in neurodevelopmental psychiatry.

